# Movement patterns of foraging common terns (*Sterna hirundo*) breeding in an urban environment in coastal Virginia

**DOI:** 10.1371/journal.pone.0304769

**Published:** 2024-07-11

**Authors:** Daniel H. Catlin, Daniel Gibson, Kelsi L. Hunt, Chelsea E. Weithman, Ruth Boettcher, Rebecca Gwynn, Sarah M. Karpanty, James D. Fraser, Shannon Ritter, Sara M. Maxwell

**Affiliations:** 1 Department of Fish and Wildlife Conservation, Virginia Tech, Blacksburg, VA, United States of America; 2 Department of Biology, University of Saskatchewan, Saskatoon, Canada; 3 Virginia Department of Wildlife Resources, Henrico, VA, United States of America; 4 Bothell Campus, University of Washington, Bothell, WA, United States of America; Hawaii Pacific University, UNITED STATES

## Abstract

Nesting colonial seabirds are prime examples of central-place foragers, animals that must return to a central location (e.g., a breeding colony) after each bout of foraging. They must balance the costs and benefits of foraging with the need to return to their colonies frequently to form pair bonds during courtship, incubate, provision mates and offspring, and protect and rear young. For some populations, the loss and degradation of suitable breeding habitat due to human activities have necessitated the construction of new breeding sites and/or the restoration of previously occupied sites. South Island, which is part of the Hampton Roads Bridge-Tunnel (HRBT) complex in the Commonwealth of Virginia, U.S.A., is a human-created island that supported Virginia’s largest mixed species seabird colony until 2020, when the expansion of the HRBT began and when all nesting seabirds were permanently excluded from the site. We studied the movement patterns of foraging common terns (*Sterna hirundo*) to determine how travel to and around foraging sites related to their colony location and to inform the siting and construction of a new breeding island. We tracked 18 individual common terns from 07 June to 29 June 2018, and we used a hidden Markov model to assign behavioral states and investigate common tern movements around the HRBT. Common terns spent more than half their time in the colony (58%), followed by time devoted to foraging (22%), and the remainder of their time was spent on outbound (15%) and inbound (5%) transit. Terns traveled as far as 98km from the colony, but on average foraged relatively close to South Island (13.6 ± 0.3km, mean ± 1 SD). Individuals tended to forage in the same locations, but there was variation among individuals. Flying to foraging sites uses energy during the already energetically costly breeding season, thus managers should prioritize placing a new colony site in a location that minimizes the distance traveled to the foraging locations frequented by the South Island birds while accounting for other life-history characteristics. These findings could help in the design and construction of new breeding sites or the restoration of current sites for other, related species, particularly for which these data do not exist.

## Introduction

Animals should balance energy expenditures in finding food with the caloric value and concentration of those resources to maximize their individual fitness [1, 2]. Thus, predators’ foraging behavior is affected by the distribution, abundance, and predictability of their prey [3]. Colonial seabirds and other central place foragers must return to a fixed location after foraging either to maintain a nest or provision a mate or young [4]. Therefore, the distribution of foraging resources can influence colony site selection, as travel time and energy spent must be weighed against the value of prey [5]. Seabirds nest in areas that balance their requirements for nutrition, nesting, individual maintenance, predator avoidance, and growth of their offspring, when possible [6].

Seabird populations and the environment in which they exist may be highly variable [7], but also may have underlying, predictable cycles [8, 9]. Birds that forage close to shore often rely on food resources that are predictable through space and time, for example exploiting prey that are only present during certain stages of the tidal cycle, whereas many species that forage offshore rely on resources (e.g., schools of fish) that may be ‘patchier’, or less predictable, in space and time [10]. Matching the spatial distribution of their prey is an efficient foraging strategy for a predator [11]. Thus, spatial variability in prey requires flexible foraging strategies [7, 9, 12], particularly if an individual is weighing both individual and offspring maintenance [13]. In these situations, individuals can assess caloric demand but must be flexible enough to withstand environmentally variable resource availability.

Another complicating factor is the loss and degradation of coastal habitats suitable for colonial nesting species, which has resulted in the decline of seabirds throughout the world largely due to human development and sea-level rise [14]. As these habitats disappear, seabirds, even common species, are increasingly reliant on restored or engineered habitats (e.g., dredge material islands) for their nesting and chick rearing [15]. Throughout the last several decades, 55 of 88 world-wide seabird habitat creation/enhancement projects were reported to have benefited breeding seabirds [15]. When restoring or creating habitat, it is important that there is suitable substrate and the right amount of vegetation for the species of interest that the area is predator and competitor-free when the target species arrive [15]. Moreover, the decision of where to focus habitat creation or enhancement efforts should take into account the location of surrounding foraging areas [16].

The Hampton Roads Bridge-Tunnel (hereafter, HRBT) is part of Interstate 64 (I-64) corridor where it crosses the mouth of the James River between the cities of Norfolk and Hampton, VA (USA). The construction of the original two-lane bridge-tunnel in 1957 created two islands, referred to as North and South islands, that served as the entrance and exit points for a single underwater tunnel that connected the low, over-water bridges on either side. A second underwater tunnel was added in 1976 which increased the number of lanes to four. In the early 1980s, South Island was colonized by common terns (*Sterna hirundo*) and black skimmers (*Rynchops niger*), presumably because the island had suitable nesting substrate, was near a plentiful food source, and was devoid of terrestrial predators [17]. Following local extirpations of several seabird colonies throughout the Chesapeake Bay and barrier islands of Virginia [18, 19], South Island became Virginia’s largest [20, 21] and most diverse seabird colony, supporting up to eight species between 2006 and 2019.

The construction of Virginia’s largest transportation initiative, the HRBT expansion project (hrbtexpansion.org) began in 2020, which resulted in the loss of South Island as a breeding site. In the spring of 2020, an adjacent artificial island and several industrial barges were successfully transformed into a temporary seabird nesting site. This site will continue to be managed for seabirds while resource management agencies work to site, plan and construct a permanent nesting island for the displaced birds [22].

In this study, we examined the behavior and movement of common terns in the area surrounding South Island to inform the siting and construction of the new nesting island. We selected common terns because they also breed in natural habitats throughout coastal Virginia, including barrier islands, shoals and saltmarshes located seaward of the Delmarva Peninsula and on a few islands and marshes in the lower Chesapeake Bay. Given the terns’ propensity for foraging in areas close to their nests [10, 23], it is likely they forage in a variety of nearshore and inshore waters, including those located in highly urbanized areas such as Hampton Roads (comprising the cities of Newport News, Hampton, Norfolk, Virginia Beach, and Chesapeake, VA). Another reason we selected common terns for our study is because they are considered a Species of Greatest Conservation Need in Virginia [24], owing to a substantial decline in the breeding population between 1993 and 2018 [21]. Thus, they are an important indicator of where to construct the new island in relation to their foraging areas.

Our objectives were to determine the areas where common terns breeding on South Island foraged, and to measure the repeatability of these choices within and among individuals. Employing technologies that are newly available for smaller seabird species [25–27], we attached GPS transmitters to a subset of incubating common terns. Common terns are visual predators, and despite a lack of formal surveys, they appear to feed within 20km of their colonies, sharing incubation between the male and female terns [28]. We hypothesized that common terns would focus their movements near the colony like other central place foragers [4], they would use predictable patterns to locate prey, and regularly exploit the same foraging locations [7]. The information on local movements and foraging locations will be used to site a new engineered seabird island. Moreover, these results and techniques could be applied in other, similar situations.

## Methods

### Study area

**Fig 1:**
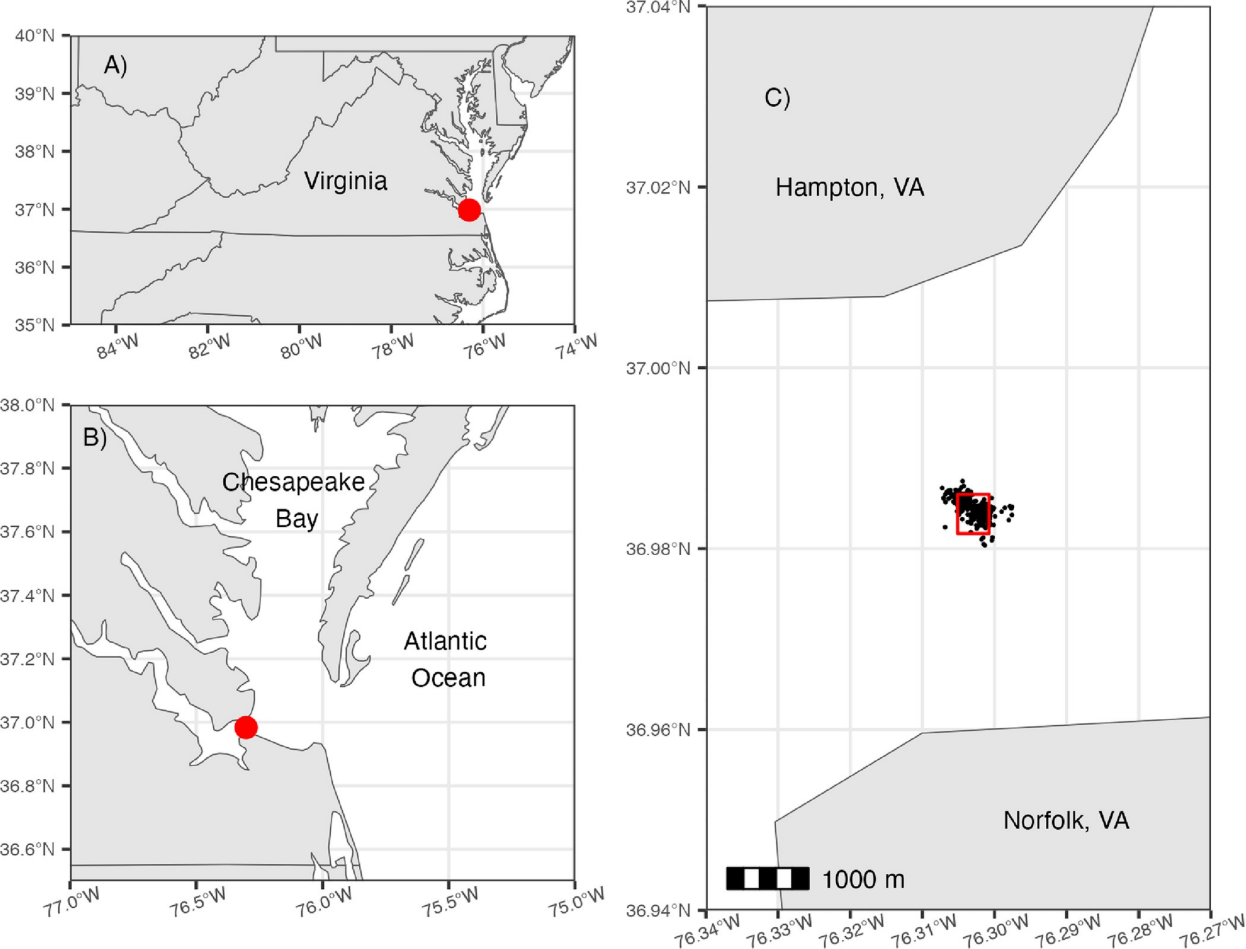
Study area map. Map of the study area A) regionally, B) the Chesapeake Bay near the colony, and C) Hampton Roads Bridge-Tunnel South Island in Hampton, Virginia. We marked the site of the former colony in each panel (red circle or rectangle). We used a Hidden Markov model to determine the behaviors (On Colony, Transit, Foraging, and Return) of 18 GPS tagged common terns. The black dots in panel C represent locations of birds classified as ‘On Colony.’ Made with Natural Earth. Free vector and raster map data @ naturalearthdata.com.

We studied the movements of incubating common terns that nested on South Island in Hampton, Virginia, USA (36.983567°, –76.302637°; Fig 1) during June and July 2018. South Island supported nearly 50% of Virginia’s common tern breeding population in 2018 [20, 21] along with other nesting seabirds including, royal terns (*Thalasseus maximus*), sandwich terns (*Thalasseus sandvicensis*), gull-billed terns (*Gelochelidon nilotica*), black skimmers, laughing gulls (*Leucophaeus atricilla*), herring gulls (*Larus argentatus*), and great black-backed gulls (*Larus marinus*).

### Field methods

We located nests by walking through colonies and placing walk-in traps on nests to capture common tern adults that were in early to mid-incubation. We recorded nest locations using a Garmin GPS unit (Garmin International, Inc., Olathe, KS, USA) and placed a wooden popsicle stick labeled with a unique number near each targeted nest. When a common tern entered the trap to incubate, we approached the nest from the direction of the door to encourage the individual to move towards the enclosed area of the trap. We removed the bird from the trap immediately. We issued an incoloy or stainless-steel band on one lower leg and a white plastic field readable (PFR) band with a unique 3-character code in black text on the opposite lower leg (Interrex, Lodz, Poland). Following banding and mass measurements, we deployed 1.6 g Pathtrack nanoFix miniR GPS transmitters (Pathtrack Ltd., Yorkshire, UK) on a subset of common terns we captured. The transmitters were approximately 1.4% of the arithmetic mean mass of the individuals we captured (112.1 g, range: 93.5–141.0 g). We set each transmitter to record a position every 5 minutes for the duration of deployment. We attached the transmitter to 3–4 central retrices using Tesa tape (Tesa Tape Inc., Charlotte, NC [23, 26]). After 4 days, we attempted to recapture tagged individuals, using the same methods, to retrieve the device and remove the tape from the tail feathers. We did not determine the sex of the birds that we captured.

### Ethics statement

This research was completed under authorization of the U.S. Geological Survey Federal Master Bander permit #21446, VDWR Scientific Collection and Bird Banding permit #62630, and Virginia Tech IACUC protocol #16–244. The island where the captures were made is owned by the Commonwealth of Virginia and managed by the VA Department of Transportation.

### Analytical methods

#### Modelling overview

We modeled the patterns of fine-scale movements of individual common terns in a hidden Markov model (HMM) framework using the momentuHMM package [29] in R (version 4.3.3). HMM-based approaches analyze individual movement data to decompose time-series location data (e.g., telemetry information) into its constituent elements (i.e., direction and speed of travel) and use variation in these movements with ancillary individual or environmental data (i.e., covariates) to assign individuals at a particular point in space and time to an unobservable state (e.g., behavior, life history stage). Model convergence requires certain *a priori* information regarding: 1) which behaviors were likely to be separable from one another based on the spatial and temporal resolution of the data, 2) a basic understanding of how an individual in a particular behavioral state should ‘act’ (e.g., a coarse approximation of how fast it should move or the consistency in direction an individual should be travelling during each behavioral state), and 3) a realistic mental model of how individual behaviors were related to each other (e.g., whether transitions between certain behavioral states were logically possible).

Common terns in our study, like other central place foragers [30], were ecologically constrained to transition between periods of incubation and travel to and from the surrounding foraging areas. Thus, we developed an HMM that accounted for these constraints, allowing individuals to transition among the following unobservable behavioral states: 1) on colony (‘O’), 2) outbound travel (hereafter, ‘transit,’ or ‘T’), 3) foraging (‘F’), and 4) inbound travel to nesting colony (hereafter, ‘return,’ or ‘R’). We predicted that individuals that were either incubating or simply loafing on the South Island nesting colony would be characterized by relatively small movements rates or stationarity that also were highly inconsistent in direction due to measurement error associated with the GPS device. Next, as individuals left the colony to find food (transit), their large-scale pattern of travel would be characterized by high rates of movement that were in one general direction and biased (i.e., moving in a direction away from the nesting colony). However, as their prey (e.g., silversides, *Menidia spp*. and killifish, *Fundulus spp*.; [10]*)* are mobile and respond to environmental cues themselves [7, 31], we expected the direction for each trip would be variable. Once a common tern arrived near an area of perceived resource availability (foraging), we predicted that movement rates would be characterized by moderate rates of speed in a less consistent direction as they attempt to key in on and capture prey. Lastly, following foraging bouts (whether successful or not), terns would have to return to the colony (return), characterized by relatively high speed and consistent direction (i.e., faster and more direct than the transit or foraging states). We predicted that these movements would be highly biased, as individuals would be traveling consistently towards the nesting colony.

**Fig 2:**
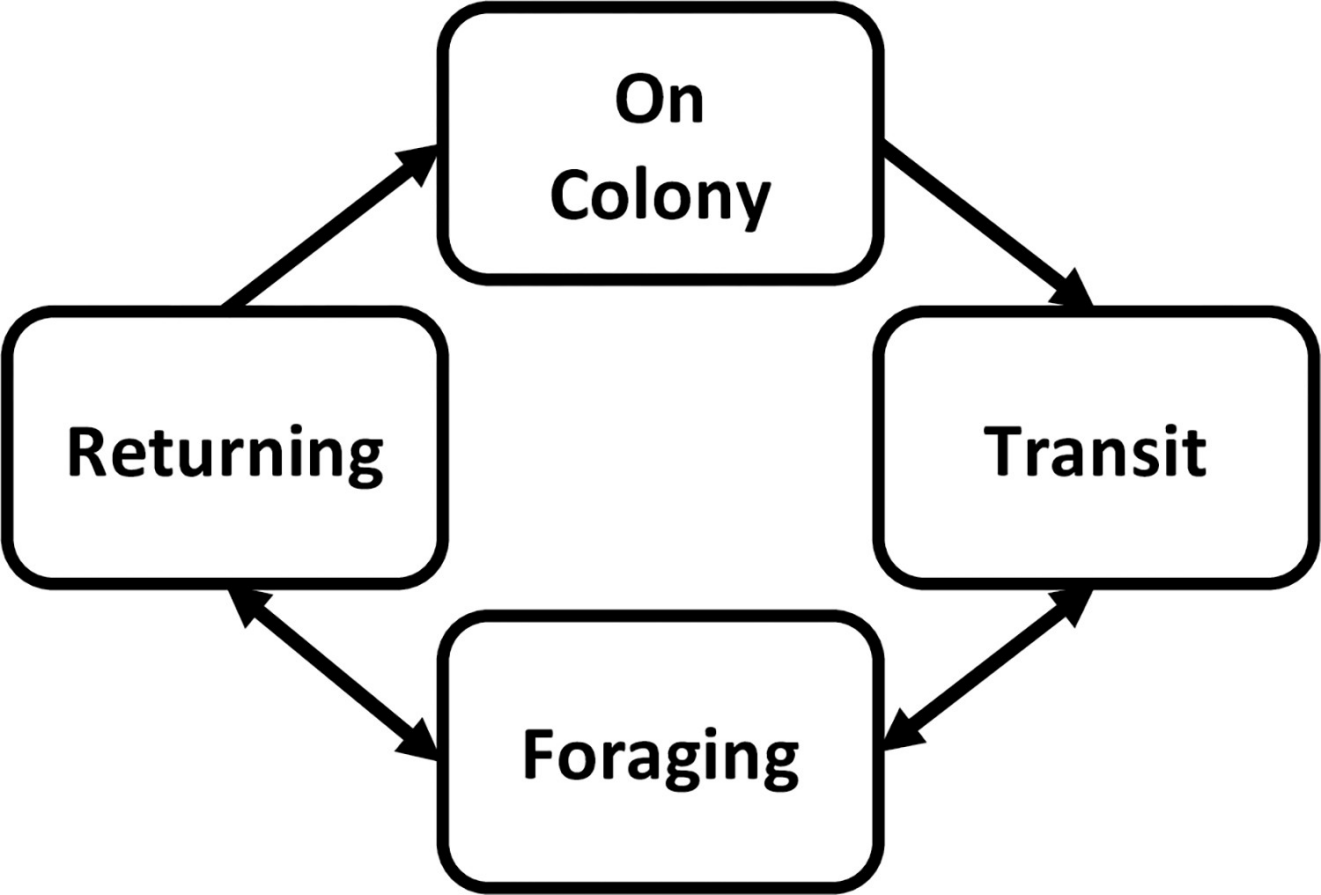
Model structure. Directed schematic of the possible behavioral states (nodes) and transitions (arrows) for the hidden Markov model used to describe patterns in nesting common tern movement behaviors. Transitions followed the direction of the arrows.

As certain state transitions were impossible (e.g., transitions from on colony to returning to colony), we constrained individuals only to travel to and from the colony in a relatively linear fashion (Fig 2). However, we allowed individuals to move freely between the transit and foraging states, as we expected that individuals may be familiar with multiple areas of food availability that would be subsequently visited until an individual was successful at foraging, or was no longer able to continue to search. We acknowledge that each of these ‘states’ most likely comprise multiple ecologically important and discrete behaviors, but further division would be difficult without additional data (e.g., altitude, dive speed).

#### Data preparation

Although each GPS transmitter was set to collect data at 5-minute intervals, some of the possible observations were missing due to transmission issues, which were exacerbated at the tail end of GPS tag deployment as the batteries were depleted. To address missing location data at the end of tag deployment, we censored all information following the third occasion that a GPS tag failed to record a location for 12 intervals in a row (i.e., an hour). For missing observations that did not meet these criteria, we interpolated the locations [29] using a continuous-time correlated random walk model [32] and assuming a bivariate normal measurement error model. The final dataset had locations for each bird for each 5-minute interval from tagging until retrieval/censoring. We simulated 250 realizations of the observed and predicted locations, and we used the pooled imputed estimates (MifitHMM function) in all later analyses [29].

#### Core model structure

The HMM framework allows a user to develop a multivariate model for all the parameters (e.g., step length, turning angle, and state transitions) and the standard deviation for step length and turning angle (see S1 Appendix for model code). We assumed step length (*l*) at each time step (*t*) followed a gamma distribution, and, for the travel states (i.e., transit and return), we modelled mean ($\mu_{t}^{l}$) and the standard deviation ($\sigma_{t}^{l}$) in step length as a function of an individual’s current distance (*cd*_*t*_) from the nesting colony. Similarly, we assumed that the directional bearing (*ϕ*) an individual was traveling followed a von Mises distribution with a concentration parameter (*ρ*), where a mean of 0 and low concentration indicates no directional bias and higher concentration values indicate lower variance around the mean or increased bias toward the mean (circular-circular regression model [29]). For the travel states, we modeled the mean angle ($\mu_{t}^{\phi }$) as a biased random walk that was a function of an individual’s bearing relative to the South Island colony (*ca*_*t*_) and the mean angle concentration parameter as a function of distance to colony. We modeled $\mu_{t}^{\phi }$ for the On Colony and Foraging states as an unbiased random walk. We constrained state transitions (*ψ*) to follow the pattern described in Fig 2. Lastly, we modeled the transit:foraging, foraging:transit, and foraging:returning transitions as a function of an individual’s current distance from the HRBT (*cd*_t_), which was used as a mechanism to allow individuals to enter the On Colony state when near the colony as well as into the foraging state as they approached local maxima. We also modeled all transition probabilities to be influenced by the time of day, using a cyclic, 24-hour consinor-based rhythmometry model [33]. This method decomposes a cycle into a linearized equation, and the user adds them to the regression in the same way as other covariates (see S1 File R code). We report covariate associations along with 95% confidence intervals and gauge support for a particular relationship based on the confidence interval overlap in a single model.

#### Spatial description of locations

To describe the location and frequency of behavioral states, we calculated a kernel density for common tern transit and foraging location points using the ‘Spatial KDE’ package in R (version 0.8). We estimated a kernel density estimator for each behavioral state. We set the grid to 100m hexagonal cells and used a bandwith of 500m to smooth the output. We used all GPS locations from all individuals, which could bias the results because of variable length deployments. With a small sample size, we were interested in the kernel estimator for illustrative purposes and acknowledge the limitations.

*Repeatability–*We quantified the proportion of the total variation in space associated with the Foraging state that was explained by within-individual level variation relative to among-individual variation. We used a nested, generalized linear mixed effects model (GLMM) to decompose the spatial variance of all model-assigned foraging locations into variance components attributed to variation within and among individuals at four levels [34]. Variation in space was represented by variation in geographical coordinates (XY) of each location assigned to the Foraging state (*o*) in which *j* = 1 and 2 represented shifts in x- and y- directions, respectively (Level 4: Observed location). For each direction (j), each observation was drawn from a normal distribution, centered on an estimated central location (*μ)*, of the trip (*a)*, with a residual variance term (*ε*^2^) (Level 3: Trip center). Likewise, the trip center (*μ*_*a*_) was drawn from a normal distribution centered on an estimated center location (*ϕ*) of an individual tern (*i*) with variance terms ($\rho_{j}^{2}$) (Level 2: Individual center). Lastly, an individual center (*ϕ*_*i*_) was drawn from a normal distribution centered on the estimated population center (*β*) with variance terms, *τ*^2^, for both directions (Level 1: Population center). The population center should resemble the geographical coordinates of HRBT, and was drawn from a broad uniform distribution that encompassed the possible coordinates. These levels are represented in the following models:

$${XY}_{o,j}∼Normal(\mu_{a,j},\varepsilon_{j}^{2})\;Level\;4:Observed\;location$$

$$\mu_{a,j}∼Normal(\phi_{i,j},\rho_{j}^{2})\;Level\;3:Trip\;center$$

$$\phi_{i,j}∼Normal(\beta_{j},\tau_{j}^{2})\;Level\;2:Individual\;center$$

$$\beta_{j}∼uniform(0,1e+08)\;Level\;1:Population\;center$$

From this model, we developed an estimate of how spatially repeatable foraging locations were, accounting for the hierarchical nature and spatial autocorrelation of the estimated foraging locations. We assumed that variation in x and y were independent from each other and estimated a shared intraclass correlation coefficient (ICC) that jointly described the proportion of the total amount of spatial variation (i.e., across both directions, sums to 1.0) in foraging locations that was explained by variation in individual-level foraging behaviors.


$$ICC=\frac{\sum \left(\tau_{j}^{2}\right)}{\sum \left(\tau_{j}^{2}+\rho_{j}^{2}+\varepsilon_{j}^{2}\right)}$$

We specified this GLMM within R with the package ‘jagsUI’ to call JAGS. For each model, we generated posterior distributions from four chains of 50,000 iterations (thin = 2) with additional adapt and burn-in periods of 25,000 iterations each. We considered models in which all parameters had Brooks-Gelman-Rubin criteria ($\hat{R}$) [35] less than 1.1 to have converged.

## Results

**Fig 3:**
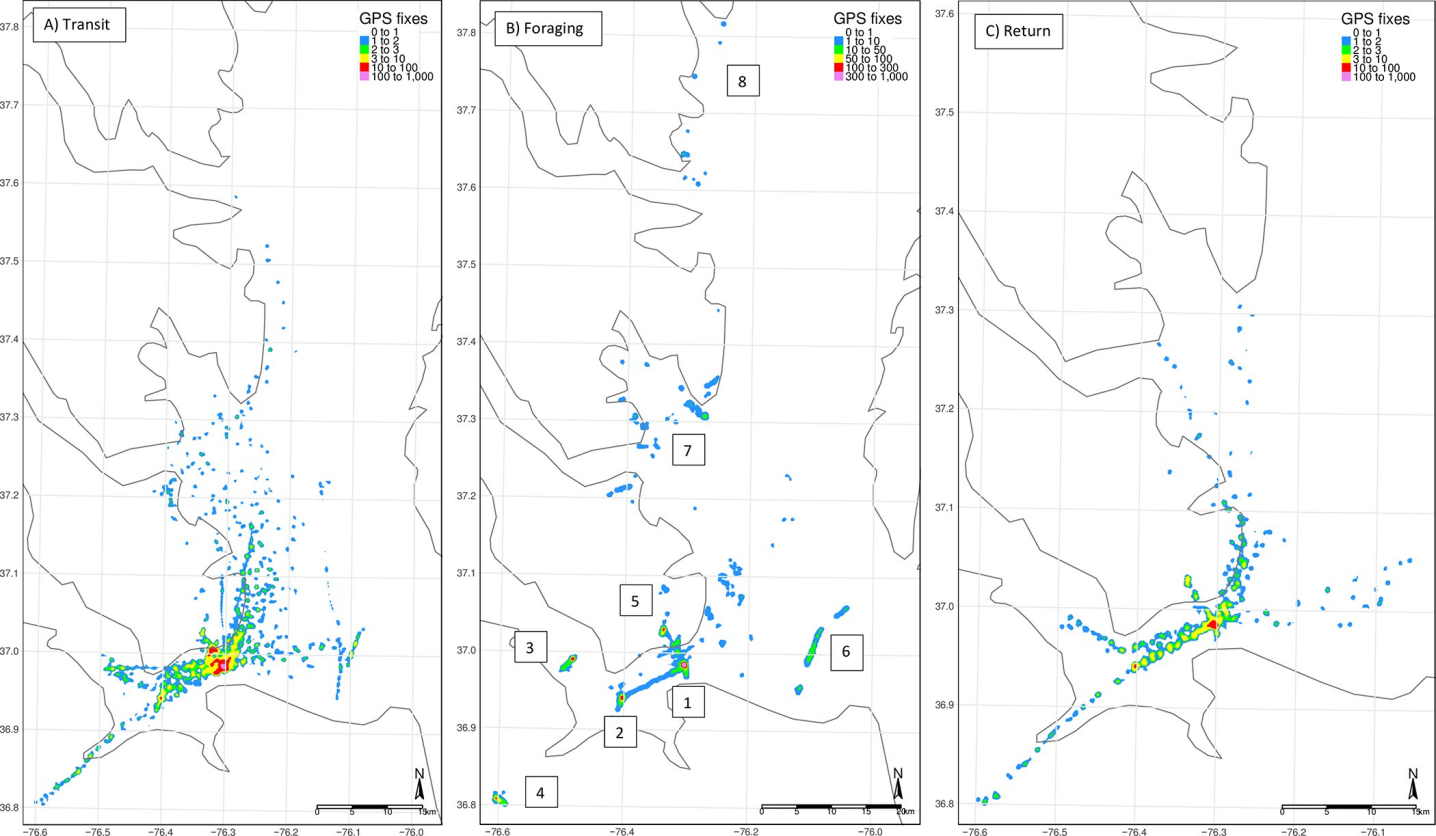
Maps of common tern GPS locations by behavioral state. A) transit from the colony to and among foraging locations, B) foraging locations, and C) return path locations. Foraging locations (B) were summarized into 8 geographical areas: 1. the mouth of the James River near the South Island colony, 2. The Monitor-Merrimac Bridge Tunnel, 3. the James River Bridge, 4. the Western Branch Reservoir, 5. the Hampton River, 6. The Chesapeake Bay Bridge Tunnel, 7. Mobjack Bay, and 8. the mouth of the Rappahannock River and Tangier Island. The kernel estimators represent a density of GPS fixes. We calculated a kernel density for common tern GPS location points using the ‘SpatialKDE’ package in R. We estimated the density for each 100m cell, setting the bandwidth = 500m. Locations and behavioral states are from a hidden Markov model. Made with Natural Earth. Free vector and raster map data @ naturalearthdata.com.

The following analyses are based on data from 18 individuals captured between 7 June–27 June 2018 (Table 1). Individuals were tagged for between 1.1–5.8d (Table 1). average Table 1). Individual locations ranged from 0.0–97.7km from the colony (Table 1), but they spent most of their time (0.43–0.71) on the colony, rather than errant (Table 1). Foraging birds were located an average maximum linear distance of 13.6 ± 0.3km (± 1 SD) from the colony in approximately 8 unique, high density (i.e., > 10 locations) geographical locations (Fig 3). The average trip distance was 46.0km, but varied among individuals (Table 1), and the overall average trip duration was 3.4h (range: 0.1–21.7 h).

**Table 1:** Individual tracking statistics for nesting common terns in Hampton, VA, USA.

Individual	Deploy	Last Location	Total Duration (d)	Prop. of Time on Colony	Total trip distance (km)	Total trip hours	Foraging trips	Trip distance (km) mean (min, max)	Trip hours mean (min, max)	Max distance from colony (km)
A	07	11	3.6	0.65	426	30.1	9	47.4 (1.9, 94.2)	3.3 (0.3, 8.1)	40.0
B	07	11	3.9	0.64	294	33.2	10	29.4 (3.8, 49.4)	3.3 (0.3, 10.3)	16.6
C	07	11	4.3	0.53	470	47.8	14	33.6 (3.9, 146)	3.4 (0.4, 18.3)	17.9
D	07	12	4.9	0.69	621	34.8	15	41.4 (0.3, 115)	2.3 (0.1, 7.5)	44.5
E	07	12	4.5	0.62	349	40.2	10	34.9 (21.4, 138)	4.0 (1.0, 20.0)	10.7
F	07	12	5.0	0.71	720	37.8	16	45.0 (5.7, 117)	2.4 (0.5, 9.3)	36.2
G	12	16	3.3	0.51	568	38.2	9	63.1 (22.7, 137)	4.2 (1.3, 13.0)	37.9
H	12	15	3.0	0.43	719	45.2	7	103 (1.7, 263)	6.5 (0.3, 18.1)	71.3
I	12	15	3.2	0.54	308	34.8	14	22.0 (0.6, 66.5)	2.5 (0.1, 9.2)	13.3
J	14	15	1.1	0.57	214	11.4	5	42.9 (5.4, 98.3)	2.3 (0.3, 5.5)	45.1
K	14	16	2.0	0.53	213	21.8	8	26.6 (5.0, 91.9)	2.7 (0.4, 7.5)	30.8
L	16	20	3.6	0.55	824	37.5	7	118 (2.6, 244)	5.4 (0.3, 9.9)	97.7
M	23	29	5.8	0.58	940	58.0	13	72.3 (2.5, 207)	4.5 (0.2, 21.7)	33.7
N	26	28	1.5	0.52	88	17.3	4	21.9 (2.4, 48.2)	4.3 (0.6, 11.5)	10.1
O	27	29	1.5	0.54	171	16.2	8	21.3 (1.0, 48.2)	2.0 (0.2, 4.1)	20.3
P	21	26	5.0	0.64	661	42.4	14	47.2 (3.2, 98.1)	3.0 (0.8, 5.5)	24.8
Q	26	29	3.2	0.58	260	32.2	7	37.2 (1.0, 78.8)	4.6 (0.2, 9.4)	19.3
R	27	29	2.0	0.51	345	22.9	8	43.2 (3.2, 104)	2.9 (1.0, 6.1)	52.5

Trips are events where an individual left the colony site and returned. Only those movements off-colony were counted in these totals. GPS locations were taken every 5 minutes until the bird was recaptured and the transmitter recovered. The last location was the last usable location prior to capture or instrument failure (see methods). Trip distance is a cumulative, path distance. The max distance from the colony is a straight-line distance. All tracking occurred in June 2018. Letters correspond with sub-panels in S1 Fig.

### State transitions

**Fig 4:**
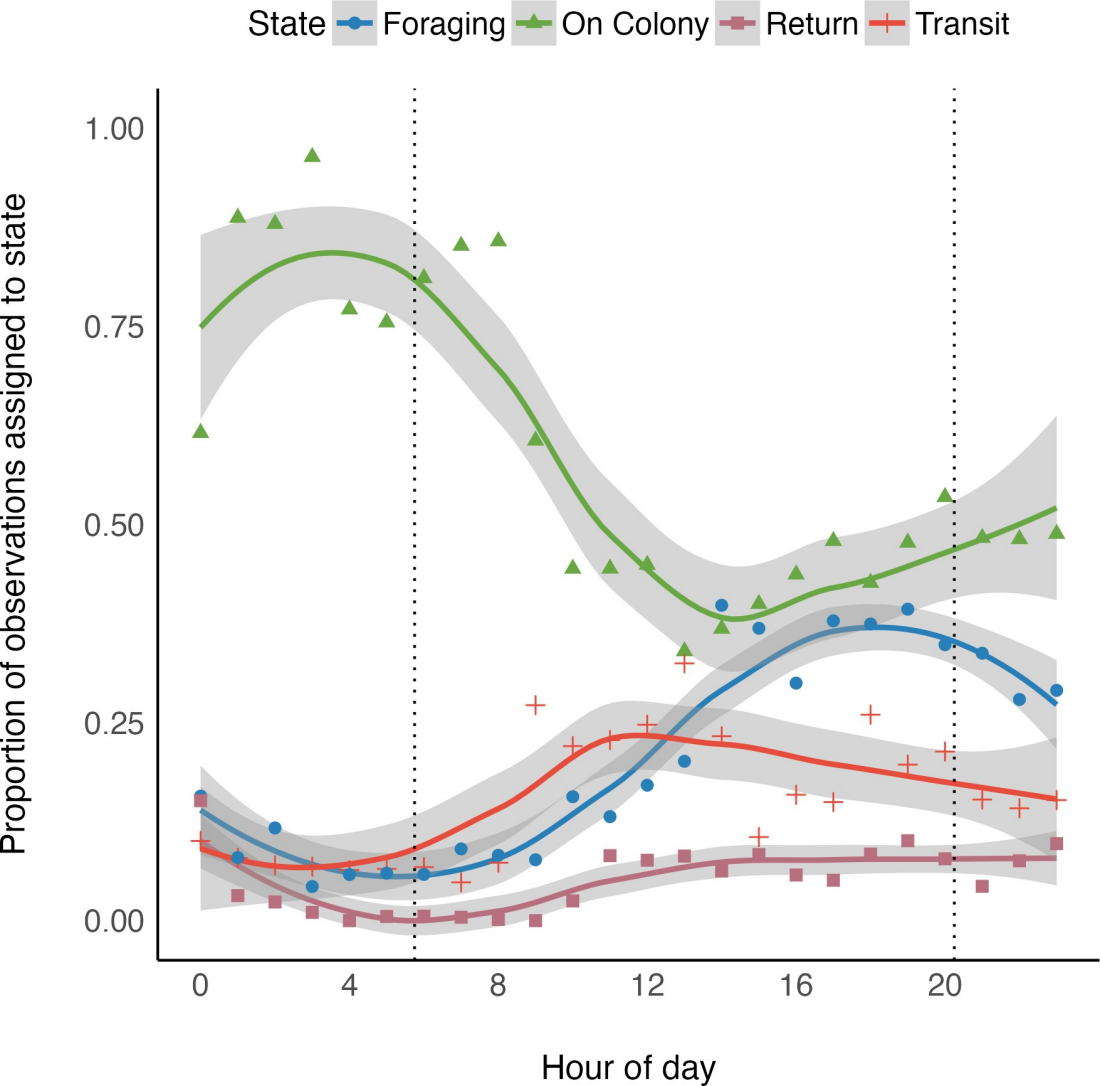
Common tern daily behavior patterns. Proportion of common terns predicted to be in each state (On Colony, green triangles; Transit, red crosses; Foraging, blue circles; and Return, maroon squares) throughout a daily cycle. Dotted lines represent the average sunrise and sunset in June for the Hampton Roads Ecosystem in VA, 2018. Estimates are from a hidden Markov model that where state assignment was correlated with distance from the colony and time of day.

Common terns spent more than half of their monitored period On Colony (*θ*_*C*_ = 0.58; 95% C.I.: 0.57–0.59), but individuals ranged from 0.43–0.71 (Table 1). The next most common state was Foraging (*θ*_*F*_ = 0.22; 95% C.I.: 0.20–0.23), followed by Transit (*θ*_*T*_ = 0.15; 95% C.I.: 0.14–0.16), and Return (*θ*_*R*_ = 0.052; 95% C.I.: 0.049–0.055). Common tern transition rates cycled throughout the day, but the probability that a tern stayed in the same behavioral state at each 5 min interval was high, regardless of time or behavior (S1 Fig), but these probabilities accumulated throughout the day (Fig 4). Daily patterns indicated that individuals predominantly waited until after sunrise (approx. 0545 hours) to leave the colony. During this daily cycle, the proportion of individuals in the Transit state increased from 0900–1300 hours (Fig 4), while peak foraging was spread from the early afternoon until late evening (Fig 4). Although individuals returned to the colony throughout the day, the highest proportion of individuals On Colony was near midnight.

Individuals were more likely to transition from the Foraging state back to the Transit state when they were farther from the colony ($\beta_{F:T}^{\psi }$ = 0.14; 95% C.I.: 0.03–0.25), but the effect was small (predicted *ψ*_*F*:*T*_ = 0.16±0.02 at the minimum distance from the colony and *ψ*_*F*:*T*_ = 0.11±0.04 at the maximum distance). We found no support for associations between Transit and Foraging or Foraging and Return relative to distance from the colony.

### Spatial description of locations

Transit and return locations were primarily within the main channel of the James River and mouth of the James River estuary, with the concentration of locations decreasing farther from the colony and the main channel (Fig 3A and 3C). The birds in this study foraged in a variety of locations spanning from Tangier Island in the northeast to Western Branch Reservoir to the southwest. There were 8 primary groupings of foraging locations around bridges and tunnels, the Hampton River, and an inland reservoir (Fig 3B).

### Speed

Average step lengths (m/5min) were substantially different among the behavioral states, with the longest being for the transit and returning states, followed by foraging and then on colony (Table 2). As distance from the colony increased, mean step length for both the Transit and Returning states increased (${\beta \mu }_{T}^{l}$ = 0.07; 95% CI: 0.06–0.08; ${\beta \mu }_{R}^{l}$ = 0.15; 95% CI: 0.13–0.18, $\mu_{T\;\min\;dist}^{l}=1636m/5min,\mu_{T\;\max\;dist}^{l}=3001m/5min,\;\mu_{R\;\min\;dist}^{l}=1990\;m/5min,\mu_{R\;\max\;dist}^{l}=6976\;m/5min$), and the variation in step length decreased (${\beta \sigma }_{T}^{l}$ = -0.12; 95% CI: -0.16–0.09; ${\beta \sigma }_{R}^{l}$ = -0.19; 95% CI: -0.27 –-0.11), which indicated that individuals were travelling relatively faster far from the colony, or relatively slower near the colony.

**Table 2:** Common tern movement statistics.

State	Step Length (m/5min)	SD Step Length	Angle (°)	Concentration
On Colony	71.5	45.9	0.00	0.00
Transit	1694.4	1062.0	0.01	2.95
Foraging	361.1	330.8	0.00	0.00
Return	2139.2	1270.6	0.05	29.06

Step length and angle of travel for each state in a hidden Markov model [29] used to describe GPS tagged nesting common tern (*Sterna hirundo*, n = 18) movement and foraging behavior in the Hampton Roads area, VA. Estimates are at the mean value for covariates (distance to colony and time of day). These models analyze individual movement data to decompose time-series location data (e.g., telemetry information) into its constituent elements (i.e., direction and speed of travel) and use variation in these movements with ancillary individual or environmental data (i.e., covariates) to assign individuals at a particular point in space and time to an unobservable state (e.g., behavior, life history stage). Positions were recorded every 5 minutes, and these data were used to place individuals into 4 states: On colony, Transit (leaving and searching for foraging areas), Foraging, and Return (returning to colony site). Angle represents the angle of travel relative to the location of the colony (0° represents the colony). Concentration is a measure of variance from a von Mises distribution where high concentration indicates less variance around the mean angle, and low concentration indicates no clear mean and high variance around it.

### Directionality

In the Transit state, individual bearing was unbiased with respect to the colony ($\mu_{T}^{\phi }$ = 0.09; 95% C.I.: 0.04–0.14, where $\mu_{t}^{\phi }$ = 0 indicates toward the colony), and with low concentration, indicating that there was no directional bias (Table 1). Bearing of travel became increasingly concentrated, or more direct, as individuals in the Transit state were farther from the colony (*β*_*ρ*_ = 0.31; 95% C.I.: 0.21–0.40, *ρ*_min *dist*_ = 2.5, *ρ*_max *dist*_ = 35.4). In the Return state, individual bearing was biased towards the colony ($\mu_{R}^{\phi }$ = 4.17; 95% C.I.: 3.06–5.27). Bearing of travel was more concentrated (Table 1) around $\mu_{R}^{\phi }$ than the transit state, becoming less concentrated (more variable) as individuals were farther from the colony (*β*_*ρ*_ = -0.28; 95% C.I.: -0.49 –-0.09, *ρ*_min *dist*_ = 33.5, *ρ*_max *dist*_ = 2.8). In the On Colony and Foraging states, *ρ* was functionally zero, indicating no concentration in direction of travel.

### Repeatability

The proportion of the total spatial variance (maximum 1) in forage locations assigned to within-individual variability, or trait repeatability, was 0.40 (95% CI 0.30–0.51). This result suggested that approximately 40% of the variation in foraging behavior was related to the predisposition of individuals to visit certain areas (S1 Fig). As less than 0.01 of the total variation was explained by within-trip variance, the remainder, or approximately 60% of the variation was associated with flexibility in foraging strategies among individuals, suggesting that foraging locations differed among individuals (S2 Fig and Table 1).

## Discussion

Common terns in this study behaved as expected for a central place forager that is tied to a nest location where it must return after each foray [4]. Terns travelled as far as 98km from the colony, but on average foraged relatively close to South Island (13.6 ± 0.3km, mean ± 1 SD). After leaving the colony, common terns spent the least amount of time in the two travel states, Transit and Return, which were presumably the costliest. Between those two states, they spent approximately 3-fold more time in the Transit (0.15, 95%CI: 0.14–0.16) state, or searching for foraging locations, than in the Return state (0.052, 95% CI: 0.049–0.055), and movement became more deliberate (higher concentration) if the birds were farther from the colony during transit. Birds in transit traveled approximately 20.3 km/h, whereas those in the return state traveled approximately 25.7km/h (Table 1). Thus, return paths were more direct and faster, which is reflected in the step lengths, suggesting that the birds were satiated, and these direct movements preserved energy as they returned to the colony. Common terns have been clocked traveling 30–50 km/h [28], which is faster than our speed measured through step length. One would expect our speeds to be lower than instantaneous speeds since step length does not account for tortuous flight paths. Nevertheless, our results were consistent with the idea that common terns were acting to minimize their energy expenditure (travel) and maximize maintenance and caloric intake [5].

For comparison, the sooty tern (*Onychoprion fuscatus*) and brown noddy (*Anous stolidus*), seabirds similar in size to the common tern, traveled 51km–973km (range of means), at speeds 15km/h (mean speed), and maximum distances from the colony: 38km–895km (range of means [25–27]). When sooty terns were food stressed, they traveled longer distances and were away from the colony longer, which may have contributed to lowered reproductive success [27]. It appeared that the sooty terns in that study adjusted their behavior to maintain their own condition (body mass did not change significantly during the study) at the cost of reproductive output [27].

If prey resources are stable or predictable, then repeated use is favored to reduce the cost of searching [7]. Individual common terns also showed relatively high repeatability in their choice of foraging locations (40% of the total variation), suggesting that knowledge of the system drove some of their behaviors. When common terns were farthest from the colony, they were more likely to switch from foraging to transit (0.16 at min distance vs. 0.11 at max distance), which could mean that they were less familiar with the locations that were farther away, thus they had to search more than when they were closer to the colony, and more familiar with the location. There were many foraging locations close to the colony, suggesting that the birds were familiar with the food resources in the area, or that they were better served by finding food close and conserving time and energy. Coupled with behavior that optimized caloric intake relative to travel, our results suggest that when a new colony is sited for common terns, managers should take the extent and location of the foraging locations in this study into consideration, ideally placing the colony near to these foraging areas without compromising other needs.

The relocation and creation of seabird colonies has become relatively common [15], both for reasons of conservation and because of land-use conflicts and resource protection [16, 36, 37]. Although relocation and siting efforts often focus on the colony site itself, off-colony food resources also can affect site selection. For example, Caspian terns in the Columbia River caused some concern because of heavy reliance on threatened and endangered salmonids [16]. In response, a historical nesting island with a greater variety of potential prey species was restored through vegetation removal. Once relocated, the Caspian terns took fewer salmonids at the new site than at their previous breeding location, and nest success was higher at the new site. Although a different situation than South Island, this example illustrates that foraging behavior is inherently a function of colony location. In our system, moving the colony from known and reliable food resources could negatively affect the success of the colony, even though the birds may be able to adjust their behavior to account for the shifts in nearby food availability [27].

Further research on common tern movement should investigate annual variability, the effects of sex, and if there are differences in these patterns relative to the stage in the breeding cycle. For example, we know that foraging behavior of common terns changes between nesting and chick-rearing [38], and parents of hatched young fed them at a rate of between 0.4 and 1.3 items per hour [10, 38]. With an average airspeed of 30–42 km/h [39], parents could make approximately 1 trip per hour at the average distance to foraging (13.6km). Thus, our results suggest that any attempt to replace the South Island colony should be within approximately 10–20 km from the original colony to accommodate common terns [28]. In addition, since these foraging locations are spread throughout the Hampton Roads area (Fig 3B), the new location should minimize the distance to each of the most frequently used foraging locations if possible.

## Conclusion

Nesting habitat for colonial seabirds is in decline, and efforts to restore and create nesting islands for them are increasing [15]. The success of these colonies depends not only on the nesting habitat, but also on the foraging resources nearby [40, 41]. Thus, studies of local foraging dynamics would benefit current and future projects aimed at colonial seabird nesting.

The seabirds at the South Island colony constitute an important part of the local ecosystem, and they rely on patchy but predictable prey proximate to their nesting location. Any attempt to relocate the breeding colony should use information about their foraging habits to improve the odds of success (i.e., acceptance of new site, breeding success). Specifically, these results create a map of regional foraging locations that can be used to site any replacement colony for this population. Even though the South Island colony has since been displaced, more studies of seabird behavior are needed to ensure that replacement colonies will fulfill the needs of the birds using them. Terns are adapted to competition among species [42], thus, it remains to be seen if our results will be applicable for the other species at the former colony site.

## Supporting information

S1 Appendix R code for analysis. Model code to analyze GPS location data using hidden Markov-models.
(DOCX)

S1 Fig Predicted transition rates across the day for nesting common terns in Hampton, VA, USA. Each facet represents the origination state, and the symbols represent the destination state. The probabilities are estimated for the mean distance from the colony for all locations (approx. 5.6 km from South Island). These values are estimated using a hidden Markov model.
(TIF)

S2 Fig Individual tracks for nesting common terns in Hampton, VA, USA. Trips are events where an individual left the colony site and returned. Only those movements off-colony are pictured. GPS locations were taken every 5 minutes until the bird was recaptured and the transmitter recovered. Letters correspond with individuals in Table 1 and numbers with 8 geographical areas: 1. the mouth of the James River near the South Island colony, 2. The Monitor-Merrimac Bridge Tunnel, 3. the James River Bridge, 4. the Western Branch Reservoir, 5. the Hampton River, 6. The Chesapeake Bay Bridge Tunnel, 7. Mobjack Bay, and 8. the mouth of the Rappahannock River and Tangier Island.
(TIF)
