# Supplementary material for: Movement patterns of foraging common terns (Sterna hirundo) breeding in an urban environment in coastal Virginia
Source: PLoS One. 2024 Jul 11;19(7):e0304769. doi: 10.1371/journal.pone.0304769 (PMC11238962; doi:10.1371/journal.pone.0304769)
Supplement: S1 Appendix — Model code to analyze GPS location data using hidden Markov-models. (DOCX) [file pone.0304769.s001.docx]

###################################################################

# The following code is associated with an analysis by Dan Catlin, an amended

# See Catlin et al. 2024 “Movement patterns of foraging common terns breeding in an

# urban environment in coastal Virginia”

# PLoS One. Information to follow.

# For information: dcatlin@vt.edu, reference COTE tracking project

# R version 4.3.3 "Angel Food Cake"

###################################################################

######################################################################

#

# These are tracking data collected from 18

# common terns that were nesting on the South Island of the HRBT tunnel.

# Full description of the model and the package. Also see momentuHMM vignette.

# https://cran.r-project.org/web/packages/momentuHMM/vignettes/momentuHMM.pdf

# McClintock, BT, T Michelot. 2018. momentuHMM:

# R package for generalized hidden Markov models of animal movement.

# Methods in Ecology and Evolution 9: 1518–1530. Doi: 10.1111/2041-210X.12995

#

########################################################################

########################################################################

#required packages. Install prior to running

library(momentuHMM)

library(jagsUI)

########################################################################

# The momentuHMM package requires a specific data type found in the following

# datafile.

load("momentuHMM.datafile.Rdata")

# Fit a (multivariate) hidden Markov model to multiple imputation data. Multiple

# imputation is a method for accommodating missing data, temporal-irregularity,

# or location measurement error in hidden Markov models, where pooled parameter

# estimates reflect uncertainty attributable to observation error.

crawl_test<-MIfitHMM(crawl_out,fit=FALSE,nSims=250,poolEstimates = F,na.rm=T,

retryFits = 10,coord = c('x','y'),centers = colony)

########################################################################

#1. ~~~~~~~~~Momentum HMM model. Hidden Markov Process~~~~~~~~~~~~~~~~~~~~~

########################################################################

stateNames <- c("colony", "transit","foraging", "return")

########################################################################

#sets up the transition matrix, NA is estimable,-100 is an impossible transition

ind_matrix <- matrix(c( NA, -100,-100, # Colony: Outbound

-100, NA,-100, # Outbound: Searching

-100, NA, NA, # Searching: Outbound & Inbound

NA,-100, -100) # Inbound: Colony

, nrow= 1)

#initial values for parameters:

Par0 <- list(step=c(50,2500,500,3500,

50,1500,200,1500)

,angle=c(0.15,0.75, 0.5, 0.75))

########################################################################

# Model:

# PSI: Distance from colony (to and from searching states)

# Step: mean: distance; SD: distance

# Angle: mean: colony angle

########################################################################

#formula for Psi.

formula2 <- ~

betaCol5(center1.distZ) +

betaCol8(center1.distZ) +

betaCol9(center1.distZ) +

betaCol1(cosinor(Hour, period = 24)) +

betaCol5(cosinor(Hour, period = 24)) +

betaCol8(cosinor(Hour, period = 24)) +

betaCol9(cosinor(Hour, period = 24)) +

betaCol10(cosinor(Hour, period = 24))

# Uses the individual matrix to constrain transitions. Betas for the other covs

fixbeta <- matrix(c(ind_matrix,

rep(NA, 12),

rep(NA, 12),

rep(NA, 12)),nrow=4, byrow = TRUE)

Par0.mod <- getPar0(model = Par0, nbStates = 4,

DM = DM,

stateNames = stateNames,

estAngleMean = list(angle = TRUE),

circularAngleMean = list(angle = TRUE),

formula = formula2)

# Model call and fitting.

MIm2 <- MIfitHMM(crawl_test$miData, nbStates=4, ncores = 8,

dist=list(step="gamma",angle="vm"),

Par0=list(step=Par0.mod$Par$step, angle=Par0.mod$Par$angle),

beta0 = Par0.mod$beta, fixPar = list(beta = fixbeta),

formula = formula2,

DM = DM,

circularAngleMean = list(angle = TRUE),

estAngleMean=list(angle=TRUE), nSims = 250,

poolEstimates = FALSE,

stateNames = stateNames)

#######################################################################

# 2. ~~~~~~~~~~~~~~~~REPEATABILITY~~~~~~~~~~~~~~~~~~~~~~~~~~~~~~~~~~~~~~~~

# Wolak, M.E., D.J. Fairbairn, and Y.R. Paulsen. 2012. Guidelines for estimating

# repeatability. Methods in Ecology and Evolution 3: 129–137.

sink("repeat.jags")

cat("

model {

for(i in 1:N) {

xy[i,1] ~ dnorm(mu.t[trip[i],1], tau.res[1])

xy[i,2] ~ dnorm(mu.t[trip[i],2], tau.res[2])

}

for(l in 1:2){

tau.res[l]<- pow(sigma.res[l], -2)

sigma.res[l] ~ dexp(1)

sigma2.res[l]<- 1/tau.res[l]

tau.trip[l] <- pow(sigma.trip[l], -2)

sigma.trip[l] ~ dexp(1)

sigma2.trip[l] <- 1/tau.trip[l]

tau[l] <- pow(sigma[l], -2)

sigma[l] ~ dexp(1)

sigma2[l] <- 1/ tau[l]

}

D.ID <- sqrt( (2 * (sigma2[1] + sigma2[2]) )/pi)

D.trip <- sqrt( (2 * (sigma2.trip[1] + sigma2.trip[2]) )/pi)

D.res <- sqrt( (2 * (sigma2.res[1] + sigma2.res[2]) )/pi)

vpc.xy <- (sigma2[1] + sigma2[2]) / ((sigma2.trip[1] + sigma2.trip[2]) +

(sigma2[1] + sigma2[2]) + (sigma2.res[1] + sigma2.res[2]))

trip.xy <- (sigma2.trip[1] + sigma2.trip[2]) /

((sigma2.trip[1] + sigma2.trip[2]) + (sigma2[1] + sigma2[2]) +

(sigma2.res[1] + sigma2.res[2]))

res.xy <- (sigma2.res[1] + sigma2.res[2]) /

((sigma2.trip[1] + sigma2.trip[2]) +

(sigma2[1] + sigma2[2]) + (sigma2.res[1] + sigma2.res[2]))

vpc.x <- (sigma2[1]) / (sigma2.trip[1] + sigma2[1] + sigma2.res[1])

vpc.y <- (sigma2[2]) / (sigma2.trip[2] + sigma2[2] + sigma2.res[2])

mu[1] ~ dunif(0, 10000)

mu[2] ~ dunif(0, 10000)

for(j in 1:nID){

mu.x[j,1] ~ dnorm(mu[1], tau[1])

mu.x[j,2] ~ dnorm(mu[2], tau[2])

}

for(k in 1:ntrip){

mu.t[k,1] ~ dnorm(mu.x[tripID[k],1], tau.trip[1])

mu.t[k,2] ~ dnorm(mu.x[tripID[k],2], tau.trip[2])

}

}

########################################################################

",fill = TRUE)

sink()

#loads the datafile required for the repeatability analysis.

load("repeatability data.Rdata")

tripID <- aggregate(as.numeric(df_1.3$ID),

by = list(as.numeric(df_1.3$Trip)), mean)

df_1.3$Trip <- as.numeric(as.factor(df_1.3$Trip))

# Bundle data

jags.data <- list(xy = (cbind.data.frame(df_1.3$x/1000, df_1.3$y/1000)),

N = dim(df_1.3)[1],

nID = length(unique(df_1.3$ID)),

ID = as.numeric(df_1.3$ID),

ntrip = length(unique(df_1.3$Trip)),

trip = df_1.3$Trip,

tripID = tripID$x, pi = pi,

center = c(0,0), R = diag(2))

###############################################

# Initial values

inits <- function(){list()}

# Parameters monitored

pars <- c('rho.id','rho.trip', 'rho.res',

'sigma', 'sigma.trip','sigma.res',

'D.ID','D.trip','D.res',

'mu','vpc.xy','vpc.x','vpc.y','trip.xy','res.xy',

'sigma2.res','sigma2', 'sigma2.trip',

'mu.x','mu.t')

# mcmc settings

n.i <- 50000

n.t <- 2

n.b <- 25000

n.c <- 4

n.a <- 25000

m3 <- jags(jags.data , inits, pars, "repeat.jags",

n.chains = n.c, n.adapt = n.a, n.iter = n.i, n.burnin = n.b,

n.thin = n.t, n.cores = 4, parallel = TRUE)
