# Supplementary figures and images for: Movement patterns of foraging common terns (Sterna hirundo) breeding in an urban environment in coastal Virginia
Source: PLoS One. 2024 Jul 11;19(7):e0304769. doi: 10.1371/journal.pone.0304769 (PMC11238962; doi:10.1371/journal.pone.0304769)

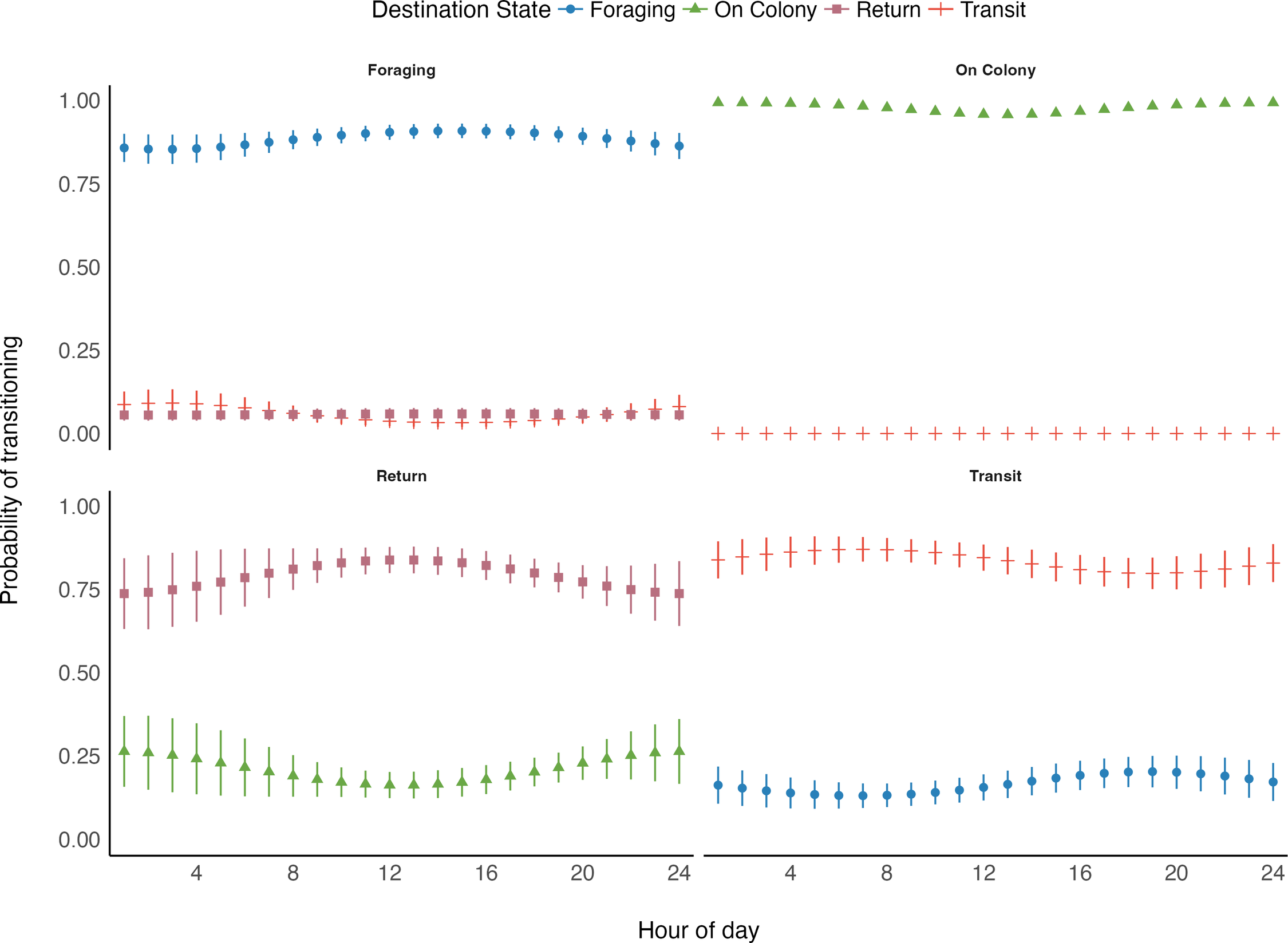

Supplement: S1 Fig — Each facet represents the origination state, and the symbols represent the destination state. The probabilities are estimated for the mean distance from the colony for all locations (approx. 5.6 km from South Island). These values are estimated using a hidden Markov model. (TIF) [file pone.0304769.s002.tif]

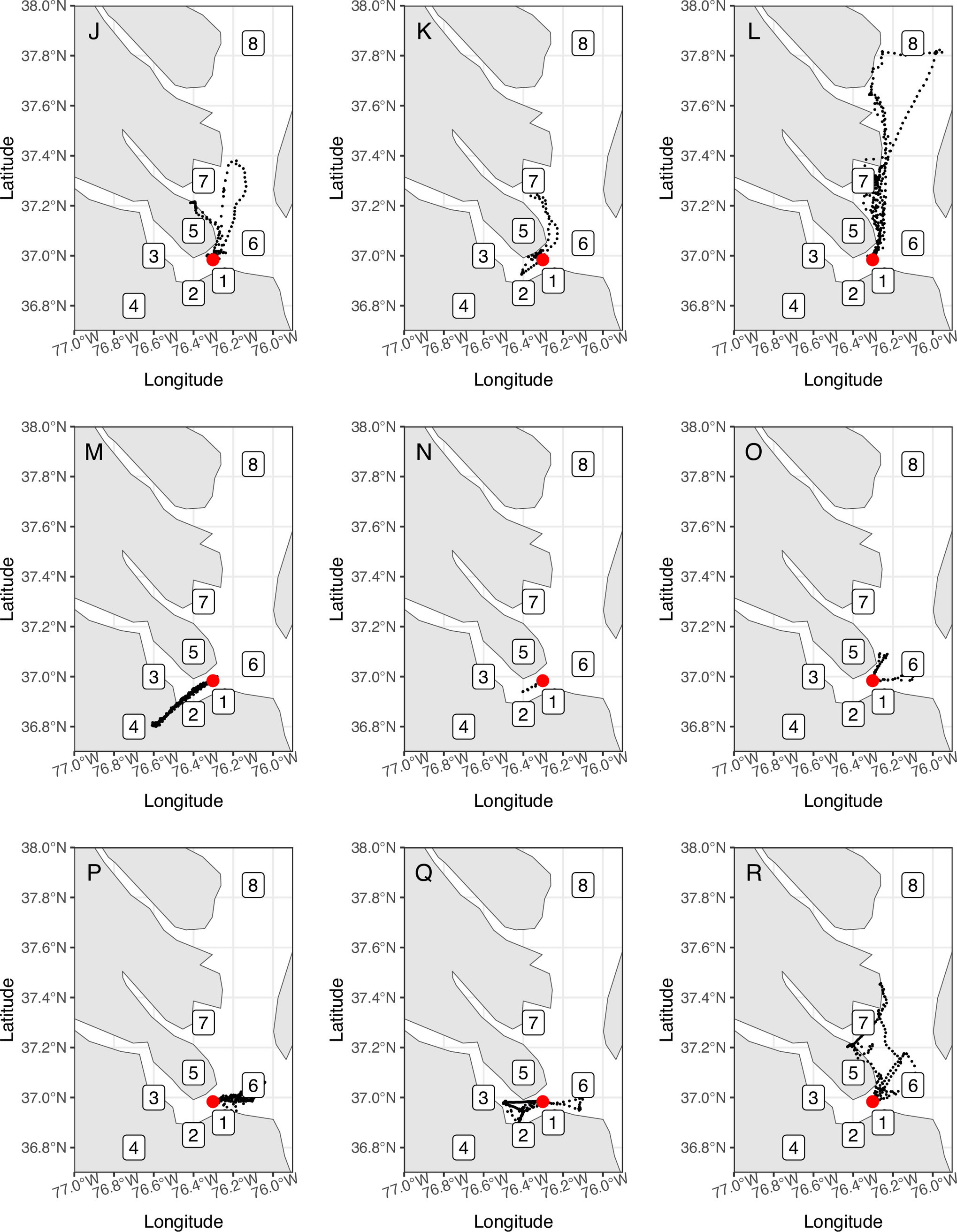

Supplement: S2 Fig — Trips are events where an individual left the colony site and returned. Only those movements off-colony are pictured. GPS locations were taken every 5 minutes until the bird was recaptured and the transmitter recovered. Letters correspond with individuals in Table 1 and numbers with 8 geographical areas: 1. the mouth of the James River near the South Island colony, 2. The Monitor-Merrimac Bridge Tunnel, 3. the James River Bridge, 4. the Western Branch Reservoir, 5. the Hampton River, 6. The Chesapeake Bay Bridge Tunnel, 7. Mobjack Bay, and 8. the mouth of the Rappahannock River and Tangier Island. (TIF) [file pone.0304769.s003.tif]
